# Bidirectional Copy–Paste Mamba for Enhanced Semi-Supervised Segmentation of Transvaginal Uterine Ultrasound Images

**DOI:** 10.3390/diagnostics14131423

**Published:** 2024-07-03

**Authors:** Boyuan Peng, Yiyang Liu, Wenwen Wang, Qin Zhou, Li Fang, Xin Zhu

**Affiliations:** 1 Graduate Department of Computer Science and Engineering, The University of Aizu, Aizu-Wakamatsu 965-8580, Japan; burrypeng@gmail.com (B.P.); liuyiyang0417@gmail.com (Y.L.); 2 Department of Obstetrics and Gynecology, Tongji Hospital, Huazhong University of Science and Technology, Wuhan 430074, China; wenwenwang@hust.edu.cn (W.W.); fangli1991@tjh.tjmu.edu.cn (L.F.)

**Keywords:** semi-supervised learning, transvaginal ultrasound, uterus perimetrium, Vision Mamba

## Abstract

Automated perimetrium segmentation of transvaginal ultrasound images is an important process for computer-aided diagnosis of uterine diseases. However, ultrasound images often contain various structures and textures, and these structures have different shapes, sizes, and contrasts; therefore, accurately segmenting the parametrium region of the uterus in transvaginal uterine ultrasound images is a challenge. Recently, many fully supervised deep learning-based methods have been proposed for the segmentation of transvaginal ultrasound images. Nevertheless, these methods require extensive pixel-level annotation by experienced sonographers. This procedure is expensive and time-consuming. In this paper, we present a bidirectional copy–paste Mamba (BCP-Mamba) semi-supervised model for segmenting the parametrium. The proposed model is based on a bidirectional copy–paste method and incorporates a U-shaped structure model with a visual state space (VSS) module instead of the traditional sampling method. A dataset comprising 1940 transvaginal ultrasound images from Tongji Hospital, Huazhong University of Science and Technology is utilized for training and evaluation. The proposed BCP-Mamba model undergoes comparative analysis with two widely recognized semi-supervised models, BCP-Net and U-Net, across various evaluation metrics including Dice, Jaccard, average surface distance (ASD), and Hausdorff_95. The results indicate the superior performance of the BCP-Mamba semi-supervised model, achieving a Dice coefficient of 86.55%, surpassing both U-Net (80.72%) and BCP-Net (84.63%) models. The Hausdorff_95 of the proposed method is 14.56. In comparison, the counterparts of U-Net and BCP-Net are 23.10 and 21.34, respectively. The experimental findings affirm the efficacy of the proposed semi-supervised learning approach in segmenting transvaginal uterine ultrasound images. The implementation of this model may alleviate the expert workload and facilitate more precise prediction and diagnosis of uterine-related conditions.

## 1. Introduction

Medical image segmentation is crucial in various diagnostic and therapeutic procedures to help clinicians make accurate diagnoses and develop treatment plans [1]. The uterine peritoneum is the outer plasma membrane of the uterus, equivalent to the peritoneum of the abdomen [2]. Similar to the abdominal peritoneum, the uterine peritoneum provides structural support and protection for the uterus [3]. Specially, the accurate segmentation of the peritoneum is critical for the diagnosis of various uterine pathologies such as fibroids, adenomyosis, and endometrial abnormalities [4]. In addition, the disruption of the uterine peritoneum can affect fertility and pregnancy outcomes. This makes accurate segmentation of the uterine peritoneum clinically important for reproductive health management. Supervised learning methods have shown effectiveness in this task [5]. However, considering the tedious and costly task of manual contour drawing for medical image annotation, semi-supervised segmentation has received increasing attention in recent years and has been widely used in medical image analysis.

In general, labeled and unlabeled data share the same distribution in semi-supervised medical image segmentation. However, in the real world, it is difficult to estimate the exact distribution of limited labeled data. Therefore, there is always an empirical distribution mismatch between the large amount of unlabeled data and the tiny amount of labeled data. To overcome this problem, semi-supervised segmentation methods always try to train labeled and unlabeled data symmetrically consistently [6,7,8,9]. Vision Mamba (VM) [10] was recently proposed for image segmentation with location-aware visual recognition through location embedding, making the model more robust in dense prediction tasks. Mamba is a new architecture for LLMs that can handle long sequences more efficiently than traditional models such as Transformers. Mamba’s efficiency comes from its bidirectional state space model, which theoretically allows for faster processing of image data compared to that of the traditional Transformer model.

In this study, we propose a bidirectional copy–paste Mamba (BCP-Mamba) for the enhanced semi-supervised segmentation of transvaginal ultrasound uterine images. The BCP-Mamba architecture can efficiently utilize the limited labeled data while leveraging the rich unlabeled data to improve segmentation accuracies. Comprehensive experiments were also performed to compare the proposed method with the well-established semi-supervised models U-Net [11] and BCP-Net [12] and demonstrate its efficacy in the segmentation of transvaginal uterine ultrasound images.

## 2. Related Work

Previous research in medical image segmentation has explored various supervised and semi-supervised learning approaches. Supervised methods typically rely on annotated data for training convolutional neural networks (CNNs) to segment the target structures in medical images accurately. However, the scarcity of labeled data poses a significant challenge in medical imaging tasks, motivating the exploration of semi-supervised approaches [13,14]. Semi-supervised learning methods aim to leverage both labeled and unlabeled data to improve model performance, often through techniques such as consistency regularization, pseudo-labeling, and data augmentation [15].

Many efforts have been made in semi-supervised medical image segmentation. Entropy minimization (EM) and consistency regularization (CR) stand out as two commonly employed loss functions. Additionally, researchers have extended the mean teacher framework in various ways. For instance, SASSNet [16] leverages unlabeled data to impose geometric shape constraints on segmentation outputs, whereas DTC [17] introduces a dual-task consistency framework by explicitly incorporating task-level regularization. SimCVD [18] explicitly models geometric structures and semantic information, constraining them within the teacher and student networks. These methods employ geometric constraints to monitor the network outputs. UA-MT [19] utilizes uncertainty information to guide the student network towards meaningful and reliable goals established by the teacher network [20]. Image-intelligent and patch-intelligent representations are combined to explore more intricate similarity cues, ensuring output consistency across different input sizes. CoraNet [21] proposes a model that generates both deterministic and indeterministic regions, with the student network assigning varying weights to regions from the teacher’s network. UMCT [22] utilizes diverse viewpoints of the network to predict the same image from different angles, employing prediction and the corresponding uncertainty to generate pseudo-labels for supervised prediction of unlabeled images.

In recent years, significant advancements have been made in the field of supervised and semi-supervised ultrasound image segmentation. Notably, Oktay et al. introduced the Attention U-Net, which enhances segmentation performance by focusing on relevant regions of the image using attention mechanisms [23]. Similarly, Li et al. presented the H-DenseUNet, a hybrid model that combines dense connections with the U-Net architecture to improve segmentation accuracy for liver and tumor segmentation from CT volumes [24]. These studies underscore the ongoing efforts to improve segmentation accuracy and robustness, providing valuable insights and methodologies that inform our approach.

These approaches significantly enhance the efficacy of semi-supervised medical image segmentation. However, they often overlook the process of learning generic semantics from labeled to unlabeled data. Treating labeled and unlabeled data separately frequently impedes knowledge transfer from labeled to unlabeled data.

Furthermore, very few studies have focused on semi-supervised segmentation of the perimetrium. This study used the BCP-Manba framework for semi-supervised medical image segmentation, which effectively utilizes unlabeled data by leveraging image translations and reconstructions. This framework is designed to correctly segment the perimetrium. The BCP-Net architecture on which it is based has on demonstrated promising results across various medical imaging modalities, laying the foundation for further advancements in semi-supervised segmentation.

## 3. Methodology

**Figure 1:**
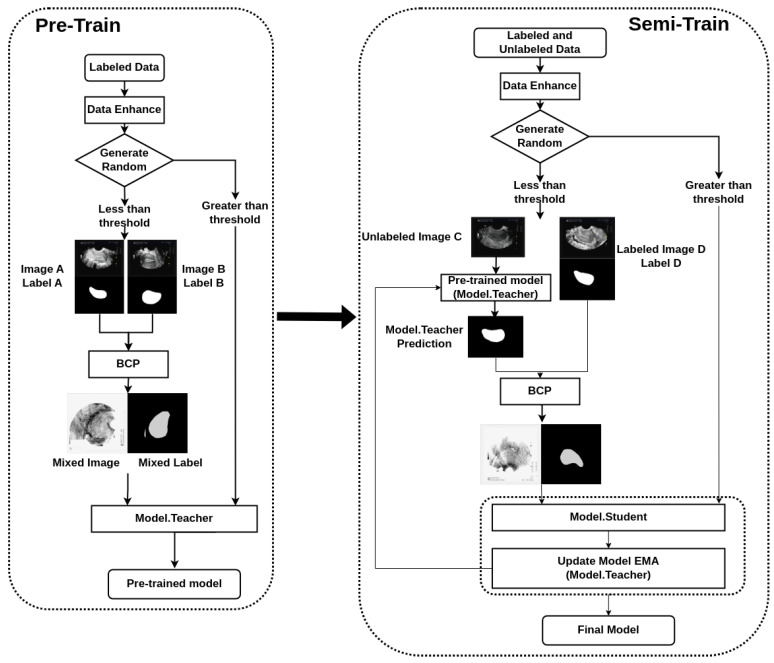
Flowchart of the bidirectional copy–paste Mamba (BCP-Mamba) model.

Our semi-supervised segmentation method is based on the BCP-Net framework. It helps to integrate labeled and unlabeled data, thereby improving segmentation performance. We have made some modifications to the BCP-Net framework. As shown in Figure 1, we added a Generate Random judgment, which enables data from different modalities to participate in the training and avoids a single input in the BCP mode. A detailed description is in the following subsection. The focus is on improving the efficiency and effectiveness of the segmentation process, making it particularly suitable for the task of segmenting transvaginal ultrasound uterine images.

### 3.1. Data Preprocessing

Before delving into the segmentation process, it is essential to preprocess the data appropriately. We start by collecting a dataset comprising both labeled and unlabeled transvaginal ultrasound uterine images. The labeled image contains manually labeled areas that correspond to the perimetrium, whereas unlabeled images lack such annotations. A total of 1940 transvaginal ultrasound images were acquired from Tongji Hospital, affiliated with Huazhong University of Science and Technology, for this study. The patient cohort encompassed individuals spanning an age range from 19 to 80 years.

### 3.2. Bidirectional Copy–Paste (BCP) Operation

The BCP operation is the core component of our methodology, and it facilitates the fusion of labeled and unlabeled images to improve segmentation accuracy. We employ a bidirectional approach, where we select foreground regions from both labeled and unlabeled images and copy them onto the background regions of the opposing images. This process allows the unlabeled images to learn common semantic information from the labeled images, thereby enhancing the segmentation performance.

Furthermore, we employ the BCP operations to generate supervised signals for training the student network. By inputting unlabeled images into the teacher network and applying dynamic soft label filtering, we generate refined pseudo-labels that guide the training process. Additionally, we introduce a confidence filtering mechanism to refine the pseudo-labels further, ensuring that only high-confidence predictions are utilized for training.

This bidirectional copy–paste technique is based on previous research and is effective in generating diverse training data by creating more realistic variations [12].

### 3.3. Experimental Details

We used 1300 images (from 152 patients) as training set, 70 images (from 10 patients) as validation set, and 570 images (from 67 patients) as test set. The training set used for pre-train included 130 images with labels, whereas that used for semi-train comprised 130 images with labels and 1170 images without labels.

Our training strategy involves several key steps. Firstly, we pre-train a model using the labeled data, establishing a baseline for segmentation performance. Subsequently, we utilize this pre-trained model to generate pseudo-labels for the unlabeled data. These pseudo-labels serve as approximations of the ground-truth masks and are crucial for leveraging the unlabeled data during training.

During each training iteration, we optimize the parameters of the network using the Adaptive Moment Estimation (Adam) [25]. Simultaneously, we update the parameters of the teacher network using an exponential moving average (EMA) [26] of the student network’s parameters. This dual-optimization process ensures that both networks learn from the available data effectively.

**Figure 2:**
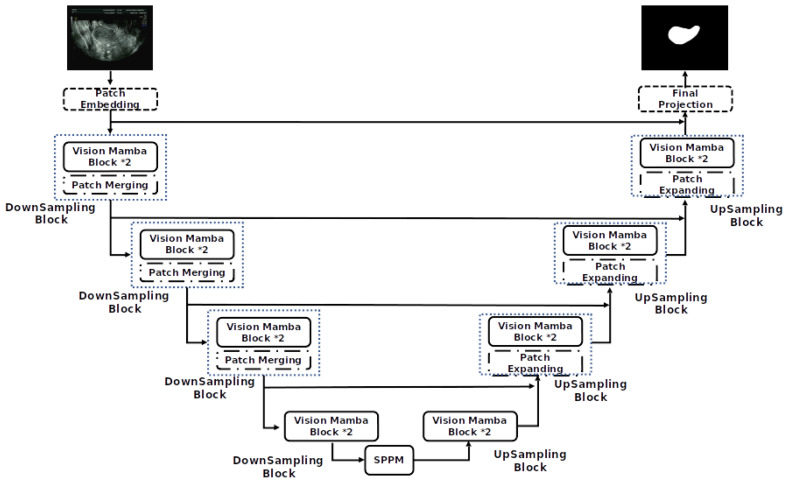
Mamba-UNet network model structure.

**Figure 3:**
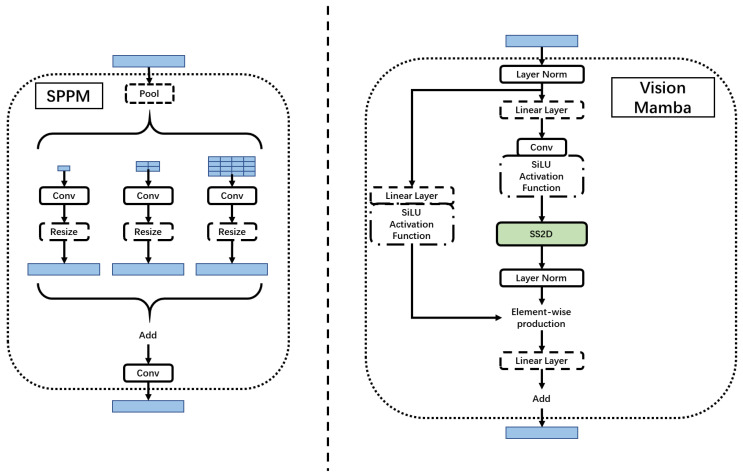
Simple pyramid pooling module (SPPM) and Vision Mamba (VM) block.

To further improve the segmentation performance, we introduce two key enhancements to the model architecture. As shown in Figure 2 and Figure 3, we first integrate the VM [10] module based on the U-Net architecture [11]. This module enhances location-aware visual recognition through location embedding, making the model more robust in dense prediction tasks. The aim of VM is to bring the cutting-edge state space model (SSM), known as Mamba [27], into the realm of computer vision.

The Mamba block is added to better segment the perimetrium of the uterus. The Mamba block has the following characteristics:

- Bidirectional modeling capability: Mamba uses a bidirectional SSM, which can analyze both forward and backward directions at the same time to model the data. This bidirectional modeling capability can better capture global contextual information and enhance the model’s performance;
- Position-aware: Mamba introduces position embeddings, which can provide spatial information perception for visual recognition tasks. This makes Mamba more robust in dense prediction tasks;
- Efficient computing and memory complexity: Compared with other SSM-based models, Mamba has higher computing efficiency and lower memory usage. It can save computing resources when processing high-resolution images and enables direct sequential visual representation learning without relying on 2D prior knowledge.

Secondly, we incorporate a simple pyramid pooling module (SPPM) [28]. As shown in Figure 3, SPPM begins by integrating input features through the pyramid pooling module, which incorporates three global-average pooling operations with bin sizes of 1×1, 2×2, and 4×4. Subsequently, the resulting features undergo convolution and upsampling operations. The convolution operation employs a kernel size of 1×1, resulting in an output channel smaller than the input channel. Following this, the features are upsampled, and another convolution operation is applied to refine them. In contrast to the original pyramid pooling module (PPM) [29], SPPM reduces the number of intermediate and output channels, eliminates the shortcut, and replaces the concatenation operation with addition. Consequently, SPPM exhibits enhanced efficiency and is better suited for real-time models.

The configuration of the system development platform is Ubuntu 18.04, an I12th Gen Intel^®^ Core™ i7-12700F×20 CPU, and an Nvidia Geforce RTX 3090 GPU.

### 3.4. Experiments

Our model was rigorously compared with the traditional supervised learning model U-Net and the original BCP-Net architecture by benchmarking our approach against these established methods to evaluate its effectiveness in semi-supervised segmentation of transvaginal ultrasound uterine images and to verify its superiority in terms of segmentation accuracy and efficiency. Specifically, we analyzed the segmentation results obtained by our model versus those of the semi-supervised models U-Net and BCP-Net on various evaluation metrics including Dice coefficient (Dice), Jaccard index (Jaccard), average surface distance (ASD), and Hausdorff_95 (HD_95) [30]. HD_95 is a metric for measuring the distance between two-point sets. It is based on the Hausdorff distance but is more robust, especially to outliers. Specifically, the Hausdorff distance measures the maximum of the minimum distances between two sets; HD_95 takes the 95th percentile of these minimum distances rather than the absolute maximum. This reduces the impact of a few outliers and provides a more stable and robust distance metric. This method is commonly used in medical image processing and computer vision tasks, especially when evaluating the performance of segmentation algorithms. Zhang et al. discussed the robustness and reduced impact of outliers when using the HD_95 metric for evaluating medical image segmentation [31]. Their research demonstrated that using the 95th percentile effectively minimizes the influence of extreme outliers while maintaining a reliable measure of segmentation accuracy. Additionally, Kamnitsas et al. highlighted the practical application of HD_95 distance in their automated brain tumor segmentation study, emphasizing its advantage in reducing the influence of outliers and providing a more stable and robust distance metric [32].

In Equations (1)–(4), *A* and *B* represent the predicted and ground-truth images, respectively, and *a* and *b* represent the pixel points in *A* and *B*. $d_{AB}$: The maximum distance from each pixel in the predicted mask to the nearest target pixel in the ground-truth image. $d_{BA}$: The maximum distance from each pixel in the ground-truth image to the nearest target pixel in the predicted mask. $d_{H}$: Hausdorff distance.

(1) $$Dice=\frac{2(A\cap B)}{A+B}$$

(2) $$Jaccard=\frac{\left(A\cap B\right)}{A\cup B}$$

(3) $$ASD\left(A,B\right)=\sum_{a\in A}\min_{b\in B}d\left(a,b\right)/\left|A\right|$$

(4) $$\begin{array}{cc} Hausdorff\_95 & =d_{H}\left(A,B\right)\\ & =\max\left\{{\mathrm{percentile}}_{95}d_{AB},{\mathrm{percentile}}_{95}d_{BA}\right\}\\ & =\max\left\{\max_{a\in A}{\mathrm{percentile}}_{95}\min_{b\in B}d\left(a,b\right),\max_{b\in B}{\mathrm{percentile}}_{95}\min_{a\in A}d\left(a,b\right)\right\} \end{array}$$

## 4. Results

The experimental results show that the proposed model outperforms BCP-Net and U-Net models in terms of segmentation accuracy. Using unlabeled data in semi-supervised training greatly improves the model’s ability to generalize to unseen data, especially in regions with limited labeled samples, and to obtain more accurate segmentation results.

As presented in Table 1, Dice and Jaccard measure the overlap between predicted segmentation and ground truth. The Dice and Jaccard values of the BCP-Mamba model are 0.8655 and 0.7762, respectively, which are higher than those of the other models. The Dice values of the BCP-Net and U-Net models are 0.8072 and 0.8463, respectively, while the Jaccard values are 0.6859 and 0.7401, respectively. Likewise, ASD and HD_95, which quantify the average and maximum differences between segmentation boundaries, respectively, are significantly lower for the BCP-Mamba model, indicating closer proximity to ground-truth annotations. The most obvious difference is with the ASD measure. The ASD values for both the BCP-Net and U-Net models are approximately 20 higher than those for BCP-Mamba. These experimental findings demonstrate the superior performance of the proposed model compared to both the BCP-Net and U-Net architectures in terms of segmentation accuracy. Despite the increased complexity of the BCP-Mamba model, the proposed model achieved a prediction speed of 73 frames per second on our device, which is comparable to the 71 and 76 frames per second achieved by the U-Net and BCP-Net models, respectively.

**Figure 4:**
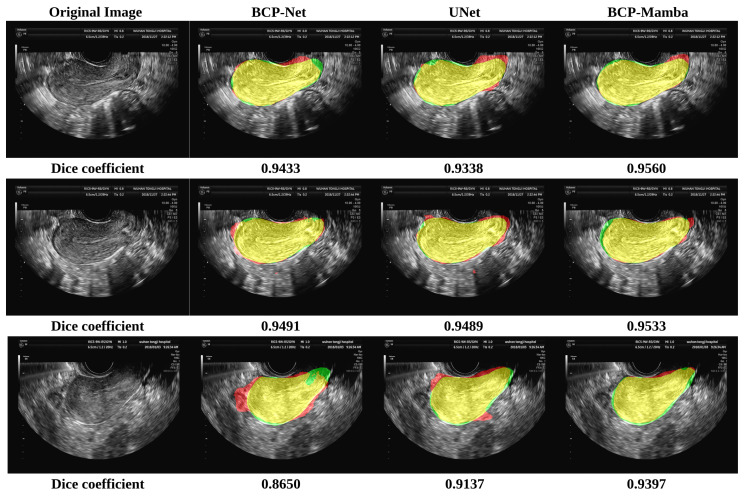
Results of the BCP-Net, UNet, and BCP-Mamba models. Ground truth (green), Predicted Results (red), and the part of overlap between the ground truth and Predicted Results (yellow).

In the comparison plot of results (Figure 4), each network has three distinct sections, indicated by colors representing the ground truth (green), Predicted Results (red), and the part of overlap between the ground truth and Predicted Results (yellow). Notably, the analysis shows that the BCP-Mamba model displays the widest area of overlap between the ground truth and predicted outcomes (yellow) compared to the areas obtained with the BCP-Net and U-Net architectures. This observation suggests that the BCP-Mamba model achieves more agreement with the ground-truth annotations, indicating higher segmentation accuracy and consistency with the underlying anatomical structures in transvaginal ultrasound uterine images.

These findings are consistent with the quantitative assessment metrics discussed earlier, suggesting the improved performance of the proposed BCP-Mamba model in accurately segmenting the plasma membrane layer.

These results underscore the significant advancements achieved by leveraging semi-supervised training with unlabeled data, enhancing the model’s ability to capture complex anatomical structures and nuances present in transvaginal ultrasound uterine images. Such improvements hold promise for advancing the diagnostic accuracy and treatment planning in uterine pathology, contributing to enhanced patient care and clinical outcomes.

## 5. Discussion

The segmentation of the perimetrium in transvaginal ultrasound uterine images holds significant clinical importance in the diagnosis and treatment of various uterine pathologies. The accurate delineation of this anatomical structure enables clinicians to assess the integrity of the uterine wall and identify abnormalities such as fibroids, adenomyosis, and endometrial disorders. Our semi-supervised approach leverages both labeled and unlabeled data to improve the segmentation accuracy, particularly in regions with limited labeled samples. By effectively integrating information from unlabeled data, our model achieves a more precise delineation of the plasma membrane layer, which is crucial for diagnosing uterine pathologies.

The utilization of semi-supervised learning techniques in medical image segmentation offers notable advantages, particularly in scenarios where labeled data are scarce or expensive to obtain. Our experimental results show that, by utilizing both labeled and unlabeled data, the semi-supervised approach enhances the generalization and robustness of the model, thereby improving the segmentation accuracy. However, the implementation of semi-supervised learning also poses certain challengess. One notable difficulty is the requirement for careful calibration of the hyperparameters and regularization techniques to prevent overfitting and ensure the effective integration of labeled and unlabeled data.

Our experimental results indicate that the proposed semi-supervised segmentation model significantly outperforms both the BCP-Net and U-Net models in segmentation accuracy. Specifically, the Dice coefficient, Jaccard index, ASD, and HD_95 metrics consistently show higher values for our model, with mean (SD) values of 0.8655 (0.0710), 0.7762 (0.1051), 40.04 (21.61), and 14.5 (8.9), respectively, highlighting its superior performance. The comparison chart (Figure 4) vividly illustrates this improvement, with the BCP-Mamba model showing the largest area of overlap between ground-truth and Predicted Results, represented by the yellow region, indicating a higher concordance with ground-truth annotations and enhanced segmentation precision.

Our approach benefits from integrating the Vision Mamba (VM) module and the simple pyramid pooling module (SPPM). The VM module enhances location-aware visual recognition, making the model more robust in dense prediction tasks, while the SPPM addresses inconsistencies due to varying image sizes. These enhancements, coupled with the semi-supervised learning framework, allow our model to effectively leverage unlabeled data, improving its generalization to unseen data and yielding more accurate segmentation results.

The reason why Mamba works in Vision tasks is that it combines bidirectional SSM, which helps model the global visual context of the data and provides location-aware visual recognition through location embedding. The advantages of Mamba include higher computational and memory efficiency, which makes it suitable for processing high-resolution images, and it performs well in ImageNet classification tasks. In addition, Mamba is more efficient, with lower GPU memory footprint and inference time, which enables it to directly perform sequential visual representation learning without relying on prior 2D information.

**Figure 5:**
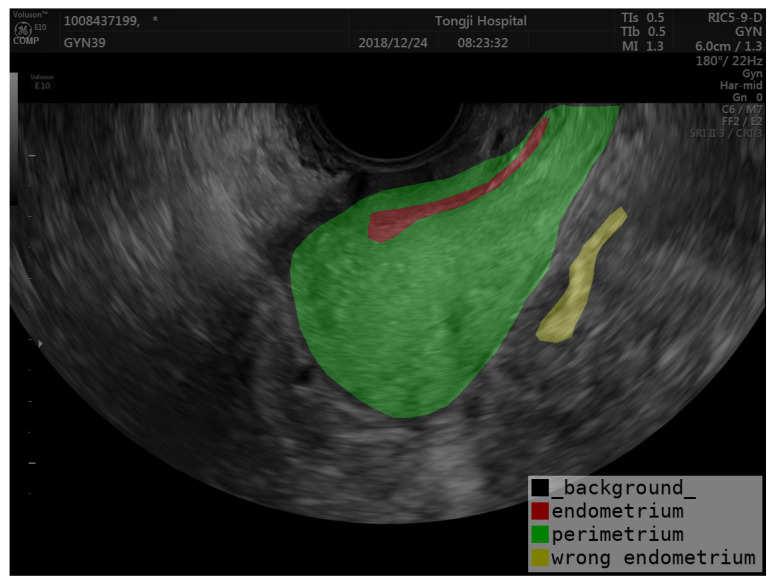
Example of endometrial prediction error.

Accurate segmentation of the uterine ectoderm improves endometrial thickness measurements, crucial for diagnosing uterine diseases. For example, in the image below, the endometrium is incorrectly categorized as being outside the uterus (marked as yellow in Figure 5) due to the uneven echogenicity of the abnormal endometrium. The correct position of the endometrium should be shown as red in Figure 5. By segmenting the perimetrium (marked as green in Figure 5) of the uterus first, we can eliminate external interference and improve the accuracy of endometrial segmentation. Our method helps reduce errors from uneven echoes and unclear boundaries, leading to better diagnostic precision and patient outcomes. The semi-supervised learning framework also increases efficiency by using both labeled and unlabeled data, reducing the need for extensive manual annotations.

A significant limitation is the scarcity of publicly available datasets specifically for transvaginal uterine ultrasound imaging. This scarcity restricts the training and validation of segmentation algorithms, leading to challenges in developing models that are both robust and generalizable. The size of the dataset used in our study was only from a medical center and relatively small, and the performance of the model may vary when applied to larger and more diverse datasets, which requires further validation and refinement. Moreover, the datasets that do exist often lack diversity in terms of patient demographics, clinical conditions, and imaging protocols. To address this limitation, we plan to collaborate with multiple medical centers to compile a more extensive and diverse dataset. This collaboration will involve both retrospective and prospective studies to rigorously test and validate our segmentation algorithms.

## 6. Conclusions

In this paper, we present the BCP-Mamba semi-supervised model for segmenting the perimetrium. Our method leverages labeled and unlabeled data to improve the segmentation accuracy, outperforming other semi-supervised models such as U-Net and BCP-Net in experimental evaluations. The satisfactory results achieved demonstrate the potential of semi-supervised learning methods in enhancing medical image segmentation tasks, especially when labeled data is limited. In addition, ectoderm segmentation technology is critical to improving the practicality of transvaginal ultrasound imaging in clinical practice and improving patient care. To ensure the extensiveness of the model, we will verify its effectiveness on other datasets in the future.

## Figures and Tables

**Table 1:** Comparison of evaluation parameters of the models.

Models	Dice (Mean (SD))	Sensitivity (Mean (SD))	Specificity (Mean (SD))
U-Net	0.8072 (0.1046)	78.74% (13.75%)	98.10% (1.27%)
BCP-Net	0.8463 (0.0979)	**94.39% (7.10%)**	96.67% (1.71%)
BCP-Mamba	**0.8655 (0.0710)**	89.82% (12.20%)	**97.96% (1.64%)**
**Models**	**Jaccard (Mean (SD))**	**ASD (Mean (SD))**	**HD_95 (Mean (SD))**
U-Net	0.6859 (0.1455)	58.59 (32.19)	23.0 (9.8)
BCP-Net	0.7401 (0.1229)	59.70 (25.87)	21.3 (10.9)
BCP-Mamba	**0.7762 (0.1051)**	**40.05 (21.61)**	**14.6 (8.9)**

## Data Availability

The datasets presented in this article are not readily available because of the restrictions of the IRB. Requests to access the datasets should be directed to W.W., petrawang@163.com.

## Abbreviations

The following abbreviations are used in this manuscript:

VSS	visual state space
BCP	bidirectional copy–paste
ASD	average surface distance
CNNs	convolutional neural networks
SPPM	simple pyramid pooling module
VM	Vision Mamba
SSM	state space model
